# Central Taxa Are Keystone Microbes During Early Succession

**DOI:** 10.1111/ele.70031

**Published:** 2024-12-31

**Authors:** Amanda H. Rawstern, Damian J. Hernandez, Michelle E. Afkhami

**Affiliations:** ^1^ Department of Biology University of Miami Coral Gables Florida USA; ^2^ Department of Ecology and Evolutionary Biology University of Toronto Toronto Ontario Canada

**Keywords:** community assembly, keystone species, soil ecology

## Abstract

Microorganisms underpin numerous ecosystem processes and support biodiversity globally. Yet, we understand surprisingly little about what structures environmental microbiomes, including how to efficiently identify key players. Microbiome network theory predicts that highly connected hubs act as keystones, but this has never been empirically tested in nature. Combining culturing, sequencing, networks and field experiments, we isolated ‘central’ (highly connected, hub taxa), ‘intermediate’ (moderately connected), and ‘peripheral’ (weakly/unconnected) microbes and experimentally evaluated their effects on soil microbiome assembly during early succession in nature. Central early colonisers significantly (1) enhanced biodiversity (35%–40% richer communities), (2) reshaped trajectories of microbiome assembly and (3) increased recruitment of additional influential microbes by > 60%. In contrast, peripheral microbes did not increase diversity and were transient taxa, minimally affected by the presence of other microbes. This work elucidates fundamental principles of network theory in microbial ecology and demonstrates for the first time in nature that central microbes act as keystone taxa.

## Graphical abstract

For decades, microbiome network theory has predicted that highly connected ‘hub’ taxa act as keystone species that disproportionately affect their communities. However, this has never been empirically tested in nature. Combining culturing, sequencing, microbial networks and field experiments, we demonstrate that central microbes significantly (1) enhanced biodiversity (35%–40% richer communities), (2) reshaped trajectories of microbiome assembly and (3) increased recruitment of additional influential microbes by > 60%.

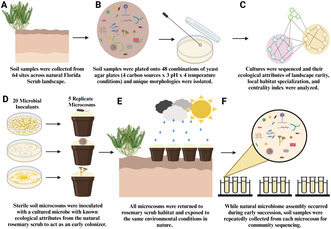

## 1 Introduction

Microorganisms drive global ecosystem processes, including decomposition, nutrient cycling, carbon sequestration and maintenance of biodiversity (Bardgett and van der Putten 2014). These benefits of microbiomes to ecosystem functions and services are ubiquitous, making it imperative that we understand which factors shape how these communities assemble. While abiotic filtering is known to be a major driver of microbiome compositional differences across distinct habitat types (Trivedi et al. 2020), intermicrobial interactions within communities also likely shape the assembly of microbiomes, especially within similar environmental/host conditions (Coyte et al. 2021). However, we know surprisingly little about which microbes are influential in structuring their communities through intermicrobial interactions, especially in free‐living, environmental microbiomes. While some attributes of microbes have been proposed to be important indicators of their influence on community structuring based on observations of natural communities—, microbial rarity/commonness across a landscape (Ortiz‐Álvarez et al. 2018) and degree of local habitat specialisation (Chen et al. 2021)—it remains untested whether these or other generalisable properties can predict which microbial taxa structure communities in nature.

For over half a century, the concept of ‘keystone species’—taxa with outsized effects on community structure and diversity (Power et al. 1996)—has been foundational to understanding community dynamics of macro‐organisms. A classic macro‐organism example of a keystone species is the Pisaster sea star, which maintains the rich diversity of benthic invertebrates and algae in marine shorelines by suppressing the dominant competitor and thereby avoiding competitive exclusion of numerous other species (Paine 1966). In addition, some keystone species are ‘ecosystem engineers’ that physically modify habitats in ways that facilitate the existence of other species in that environment. For instance, gopher tortoises excavate large subterranean burrows that also provide a shaded refuge for many other species (Kinlaw and Grasmueck 2012) and beavers build dams that reshape the local ecosystem's hydrology, thereby restructuring fish, waterfowl and plant communities (Brazier et al. 2021). This keystone species concept from community ecology could provide a valuable pathway forward for understanding influential taxa within microbiomes.

A keystone microbes' influence on early‐stage successional dynamics within microbiomes may be of particular importance as initial microbial communities have been shown to have long‐term cascading effects on the microbiome and the broader ecosystem. For example, previous research suggests that differences in microbiome composition immediately post fire (i.e., 1 day later) leads to divergent ecosystem recovery times (Johnson et al. 2023) and altered nutrient cycles (Nelson et al. 2022), and these types of changes can persist up to 20 years (Pérez‐Valera et al. 2020). Manipulations of initial compositions of field soils have resulted in depletion of native microbial species (Moreira‐Grez et al. 2019) and restructuring of soil‐borne nematode compositions (Wubs et al. 2019) lasting from months to decades. Additional research has shown strong relationships between early microbiome compositions and future plant germination rates (Eldrige et al. 2021), resilience to pathogens (Wei et al. 2019), tolerance to herbivory stress (French, Kaplan, and Enders 2021) and prediction of crop quality (Asad et al. 2023). Because microbiomes are hyperdiverse and interactions among microbes cannot be directly observed within most natural environments, identifying the microbial keystone species that structure these early communities presents a particularly daunting challenge.

At the centre of an ongoing debate over the last decade is whether network properties can predict which microbes are the influential, keystone species within microbiomes. Central taxa—taxa that are highly connected within microbial networks—have been theorised to be keystone species that disproportionately structure microbial communities (Banerjee, Schlaeppi, and van der Heijden 2018). Current work supporting central microbes as keystone species is based on computational predictions (Berry and Widder 2014; Trosvik and de Muinck 2015), synthetic communities (Niu et al. 2017; Venturelli et al. 2018) and laboratory manipulations (Agler et al. 2016; Xun et al. 2021), but no studies have experimentally tested whether central taxa govern microbiome community dynamics in nature.

To address this gap, we combine culturing, sequencing and microbial networks to isolate ‘central’ (highly connected, hub taxa), ‘intermediate’ (moderately connected) and ‘peripheral’ (weakly/unconnected) microbes and use field experiments to experimentally evaluate their effects on soil microbiome assembly during early‐stage succession in nature. We then monitored community properties to see which attributes of the experimental early coloniser best predicted their effect on community assembly. This integrative research revealed that central microbes are keystone species in nature, with these taxa substantially enhancing biodiversity, predictably structuring community composition and increasing recruitment of other influential microbes into assembling communities during early succession.

## 2 Methods

### 2.1 Study System

This study was conducted in the endemic Florida rosemary scrub at Archbold Biological Station (Venus, FL, USA). The rosemary scrub soil microbiome is distinct from the surrounding flatwoods habitat (Hernandez et al. 2021) and is important for the persistence of imperilled plant populations (David et al. 2019). This ecosystem is naturally pyrogenic where fires sterilise the top layer of soil (Carrington 2010), necessitating microbiome reassembly from nearby unburnt patches (Revillini et al. 2022). Recent research from the same sites used in our study showed that plant species that were able to tolerate/benefit from early successional microbiomes were most successful at establishing (Revillini et al. 2022). Due to the habitat's importance, distinct microbiome and the disturbance effects of fire, this is an excellent system for studying how attributes of early microbial colonisers affect microbial community succession.

### 2.2 Characterisation of Rosemary Scrub Taxa's Ecological Attributes

**FIGURE 1:**
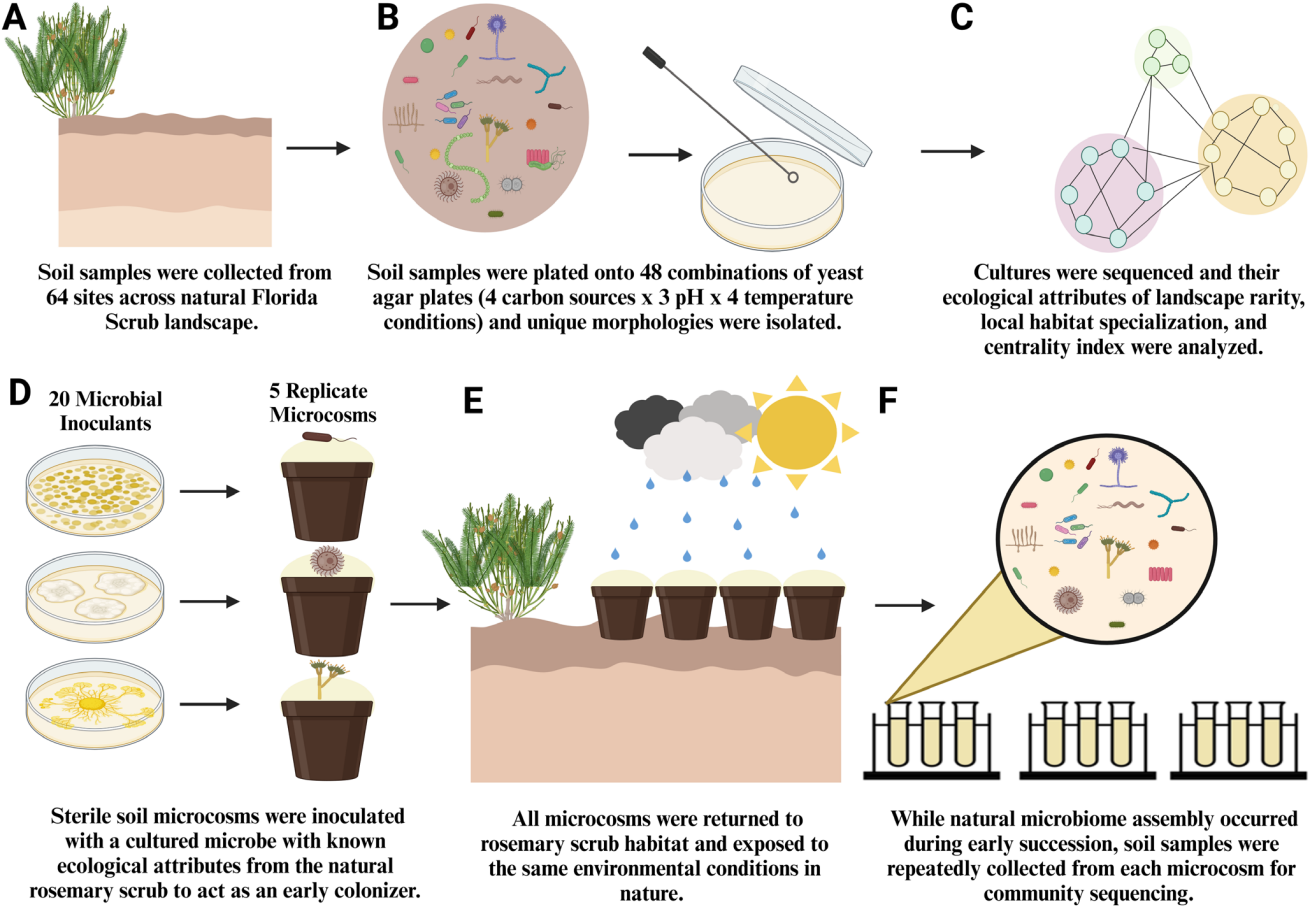
Overview of the design for the manipulative field experiment. (A) Soil samples were collected from 64 rosemary scrub patches across the natural Florida Scrub landscape. (B) To increase diversity of our culture collection, we varied the growth conditions in a factorial design with 4 carbon sources (mannitol, glucose, sucrose, maltose), 3 pH levels (6, 7, 8) and 4 incubation temperatures (23°C, 28°C, 37°C, 55°C). (C) We matched our isolated taxa to the natural microbiome by comparing the 16S sequences of the cultures to the community‐wide NGS‐generated sequences from across the rosemary scrub community using BLAST. We characterised our cultures in three ways: Their landscape rarity in the ecosystem (i.e., rosemary scrub occupancy), their degree of local habitat specialisation and their centrality index within the microbiome network. (D) Microcosms were created by inoculating sterilised field soil with 5 mL of microbial inoculum from one of 20 sequenced and identified isolates from the Florida Rosemary Scrub to serve as experimental early colonisers or 5 mL of sterile water as a control (21 treatments × 5 replicate microcosms = 105 total microcosms). The inoculum level (1 × 10^4^ cells per g of soil) represented ~1% of the microbial abundance in typical rosemary scrub soil and a biologically relevant abundance for a single early coloniser species in a natural habitat after fire. (E) Microcosms were deployed in a completely randomised design back into the Florida Rosemary Scrub habitat within an open sand patch at least 0.5 m away from allelopathic Florida rosemary shrubs ( *Ceratiola ericoides* ) found at patch edges. The bottom of the microcosms were shallowly buried in the soil to allow microbial migration from below as well as from the air. All microcosms were exposed to the same environmental conditions (i.e., natural weather conditions) for the duration of the experiment. (F) Soil samples were collected across three time points during early succession (1, 7 and 14 days after being placed in the field) for amplicon sequencing and diversity, richness, composition and migration of taxa of each assembling prokaryotic community was determined. This figure was created using a licence from BioRender.com.

**FIGURE 2:**
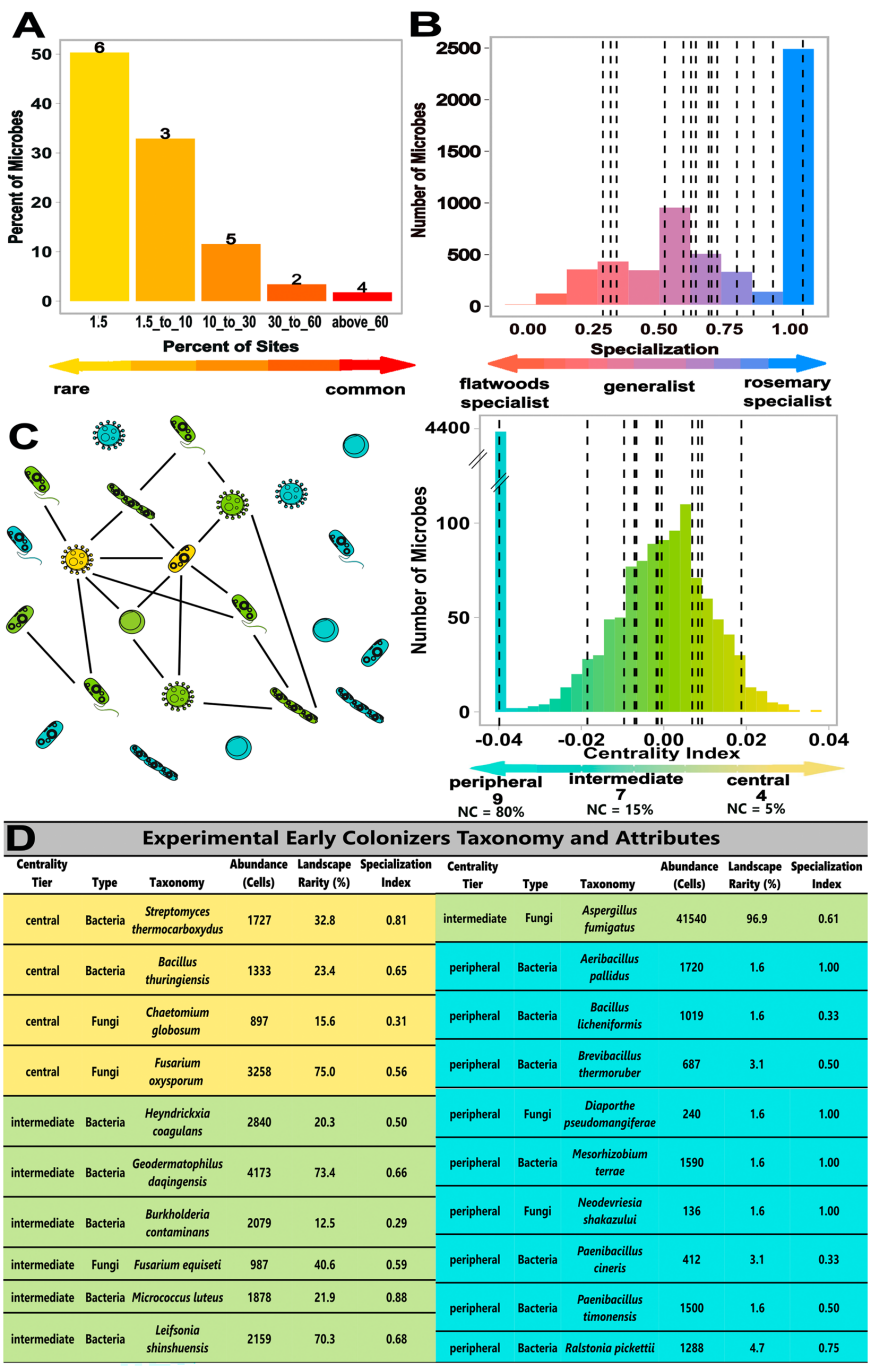
Ecological attributes of isolated microbial taxa used in the field experiment span a wide range of natural variation within the microbiome. (A) Landscape rarity was measured as the percentage of rosemary scrub patches occupied across the landscape. (B) Local Habitat specialisation on the rosemary scrub was measured as the number of rosemary scrub sites occupied divided by the number of rosemary and flatwood sites occupied (ranging from rosemary specialist (Specialisation Index = 1) to non‐specialist (SI = 0.5), with a few isolates somewhat specialising on the other habitat type (SI < 0.5)). (C) Centrality Index—how connected each taxa is within the microbial network—was determined by conducting a principal component analysis of 4 centrality metrics (degree, betweenness, closeness, and eigen centrality) and extracting the PC value from the primary axis of variation. The schematic (left) is a diagrammatic representation of a microbial co‐occurrence network with highly connected ‘central microbes’ in yellow, ‘intermediate microbes’ in green, and ‘peripheral microbes’ in blue. Peripheral microbes were assigned the minimum centrality index since these taxa fall outside the network. NC denotes the percentage of the whole community microbes that fall into each centrality category. For panels A‐C, bars represent the microbes in the overall natural rosemary scrub community that fall within each bin (based on our independently collected NGS data set across 64 sites). Bold numbers or dashed lines indicate unique isolated taxa (biological replicates) selected as “early colonisers” in the experiment that fall within each bin. Overlapping dashed lines indicate that multiple early colonisers shared the same attribute for that category. (D) Taxonomy, average soil abundance, and ecological attributes of experimental early colonisers. Taxonomy was determined by selecting the top NCBI BLAST identity match to the culture's 16S or ITS region while average abundance was the average number of cells per gram of soil across all patches in which each culture was found in the rosemary scrub.

Using NGS‐generated whole community microbiome data (Hernandez et al. 2021) for soil crusts from 103 Archbold sites (64 rosemary scrub patches and 39 adjacent flatwoods habitats; Figure 1A), we performed new analyses to characterise three ecological attributes of all microbial taxa within the broader rosemary scrub microbiome (Figure 2A–C).

First, we determined landscape rarity by calculating the proportion of all sequenced rosemary scrub patches (64 patches) that the microbe occurs in.

$$\text{Landscape rarity}=\frac{\mathrm{No}.\;\text{of rosemary sites microbe occupies}\;}{\mathrm{No}.\;\text{of total}\;\text{rosemary sites}}.$$

Second, we determined the local habitat specialisation (to rosemary scrub) by calculating the relative frequency with which the microbe occurred in rosemary scrub habitat compared with neighbouring flatwoods habitat (David et al. 2020). This index ranges from 0 (flatwoods specialist) to 1 (rosemary scrub specialist) where 0.5 is a generalist.

$$\text{Specialization}=\frac{\mathrm{No}.\;\text{of rosemary sites occupied}}{\mathrm{No}.\;\text{of rosemary AND flatwoods sites occupied}}.$$

Third, we assessed centrality in the broader rosemary scrub microbiome network as a measure of how connected a node (here a microbial taxon) is within the community using a composite centrality index. The index incorporated four commonly used centrality metrics: (1) degree centrality (how connected a node is to other nodes in the network; Proulx, Promislow, and Phillips 2005), (2) betweenness centrality (how often a node lies on the shortest path between all other nodes; Koschützki and Schreiber 2008), closeness centrality (how close a node is to all other nodes; Freeman 1978) and eigen centrality (how often a node is connected to other nodes that are also central; Ruhnau 2000). Each metric was calculated using Networkx (v3.3; Hagberg et al. 2008) within Python (v3.10.14) (File S1). Then, the composite centrality index was determined by conducting a principal component analysis of all centrality metrics and extracting the PC value from the primary axis of variation (i.e., axis that explained ~98% of variation; Figure S1). All individual centrality metrics were highly positively correlated with one another (*r* value range: 0.92–0.99; Figure S2), which is common in many networks (Valente et al. 2008). Therefore, a higher centrality index value denotes a microbe with greater overall connectivity to other microbes.

The network used in the calculations above was a *denovo* clustered (Westcott and Schloss 2015) cross‐domain microbiome network (Tipton et al. 2018) constructed using SparCC, which accounts for sparsity commonly found in compositional data (Friedman and Alm 2012). The network used 97% sequence similarity OTUs present in ≥ 10% of all sites with default parameters (Ma et al. 2020) and proportional relative abundance to normalise and combine 16S and ITS data (File S2). Taxa were binned into centrality tiers (Ma et al. 2016) using the following criteria: The top 5% of all microbes with the highest centrality index (centrality index range: between 0.037 and 0.007) were classified as central (predicted keystones), the other microbes in the top 20% of centrality index were classified as intermediate (centrality index range: between 0.007 and −0.04), and the remaining microbes (bottom 80% of centrality index) were considered peripheral microbes (i.e., transient) (File S3; Figure S1). Peripheral microbes were assigned the minimum centrality index (−0.04) since these taxa fall outside the network. To evaluate the robustness of our conclusions about the role of centrality in microbial community assembly from our field experiment (described below), we also conducted four follow‐up analyses testing consistency with different construction methods (please see Supplementary Methods: *Evaluating robustness of network construction* for details).

We then identified these same three attributes for a collection of taxa we isolated from Archbold soils (Figure 1; please refer to Supplementary Methods: *Isolation and identification of rosemary scrub microbial taxa* for details) by comparing Sanger sequences of the cultures to NGS‐generated sequences from across the rosemary scrub community using BLAST (Madden 2005) (Figure 1B,C). We selected 20 unique taxa to use as experimental ‘early colonisers’ in our field experiment (described below). These taxa were selected because they spanned the natural community distributions of the three attributes tested (Figure 2A–C), represented a diversity of taxonomies (Figure 2D) and had‐high quality sequencing data that reliably matched to the microbiome‐wide data using the standard *e*‐value criteria (*e*‐value < 1 × 10^−50^; Madden 2005). To distinguish between top BLAST hits with similar quality (i.e., *e*‐values), we chose the top hit found in at least 10% of the sampled field sites as the isolated taxon's identity. To determine the original soil abundance of each early coloniser, we used previously collected cell count qPCR data from the rosemary scrub (Hernandez et al. 2021) multiplied by the relative abundance to estimate absolute cell count abundances per gram of soil.

### 2.3 Field Experiment

To assess how characteristics of early colonising microbes influence succession, we set up a manipulative field experiment where we monitored early microbial community assembly in soil microcosms inoculated with each of our 20 isolated taxa (as well as uninoculated microcosms) at Archbold Biological Station (Figure 1D). Inoculated microcosms ultimately contained 1 × 10^4^ cells per g of soil, which is approximately 1% of the microbial abundance in typical rosemary scrub soil samples (Hernandez et al. 2021). This inoculum amount represents biologically relevant levels of abundance for an individual early coloniser species in a natural habitat after a severe fire (Ammitzboll et al. 2021). (Please see Supplementary Methods: *Set‐up of experimental microcosms* for details).

Inoculated microcosms were wrapped in sterile foil and immediately transported to Archbold Biological Station rosemary habitat patch ‘1 N’ (Latitude 27.20, Longitude −81.36). Microcosms were deployed in a completely randomised design within an open rosemary scrub sand patch (Figure 1E). We collected 315 soil samples in sterile 2‐mL tubes across three time points during early succession (1, 7 and 14 days after being placed in the field, Figure 1F). Two adjacent microcosms out of the 105 were removed from the study due to evidence of animal disturbance. In addition, to characterise the source pool of the assembling microbiomes, we sequenced 12 soil samples from the nearby rosemary scrub. All soil samples were stored at −80°C until DNA extraction.

DNA was extracted from each soil sample using the DNeasy PowerSoil Pro QIAcube HT Kit following modified protocols (Revillini et al. 2022). Prokaryotic 16S libraries were sequenced on an Illumina MiSeq Sequencer (v3, 300 bp paired end), processed through QIIME2 (Bolyen et al. 2019) (v.2022.2), and replicates were pooled and rarefied (to 6000 reads). (Please refer to Supplementary Methods: *Microbiome DNA extraction and sequencing* for details).

### 2.4 Microbial Diversity, Composition, and Connectivity Analyses

Prokaryotic Shannon diversity, richness, and evenness of the microcosms were calculated in the R package *vegan* (v2.6–4; Oksanen et al. 2020). To determine which early coloniser attribute(s) affected community diversity, model selection was employed using the *dredge* function (R package *MuMIn* v1.47.5; Kamil 2021). The fixed effects terms considered in the full model were landscape rarity, specialisation index and centrality index of the experimental early colonising microbe the microcosm received, and the random effect was time point of microbiome collection within treatment group. The model with centrality of the early coloniser as the only predictor was the best model based on lowest delta AICc (Arnold 2010; Figure S3). The estimated proportion of variance explained by the fixed effects in the model was calculated using a Spearman correlation.

After finding centrality was the most important attribute predicting prokaryotic diversity and richness, we conducted follow‐up analyses on how this attribute affected the composition of the community. To do this, we conducted a distance‐based redundancy analysis (db‐RDA) using Jaccard distances, which is recommended for its high sensitivity in detecting differences in presence/absence between microbiome groups (Kers and Saccenti 2022) (using *capscale* in R package *vegan* v.2.6–4; Oksanen et al. 2020). The terms used in the db‐RDA were centrality and time. Repeated measures PERMANOVA was used to assess whether the centrality gradient was significant across time points (using *adonis2* in *vegan*).

To understand how early coloniser centrality impacted migration of other microbes into the assembling communities, all the ESVs from the assembling microbiomes within our experimental microcosms were matched using *BLAST* to the OTUs from our rosemary scrub microbiome network. This allowed us to extract the centrality metrics of each microbe that migrated into the microcosms. Taxa from the assembled communities were then binned into centrality tiers using the same method employed earlier. The mean migration rates for central, intermediate and peripheral microbes into the assembling communities were each calculated using the formula below.

$$\text{Mean migration rate}=\frac{\mathrm{No}.\;\text{of taxa observed in community}}{\text{Total number of days}}.$$

ANOVAs with *post hoc* Tukey's HSD tests were performed to compare the mean migration rates of the microbes between microcosms inoculated with early colonisers with different centrality tier attributes.

## 3 Results

### 3.1 Ecological Attributes of Experimental Early Coloniser Microbes Span a Wide Range of Natural Variation Within the Microbiome

The 20 cultured microbes selected as experimental early colonisers spanned a wide range of the natural variation across all three ecological attributes tested (99.9% of the landscape rarity, 91.6% of the local habitat specialisation and 99.2% of the network connectedness of natural distributions across 64 field sites; Figure 2), making them excellent representatives of the natural community. These microbes were also widely taxonomically distributed across 17 distinct genera, spanning both fungi and bacteria (Figure 2D). We found that central taxa within the network had both stronger net positive strength and net negative strength than intermediate taxa (positive: *R* = 0.21, *p* < 0.001; negative: *R* = −0.21, *p* < 0.001, Figure S4A,B), suggesting that central microbes may facilitate increases in diversity through a combination of stronger positive and negative interactions with other microbes in the community.

### 3.2 Central Early Colonising Microbes Increase Diversity and Richness in Early Successional Communities

**FIGURE 3:**
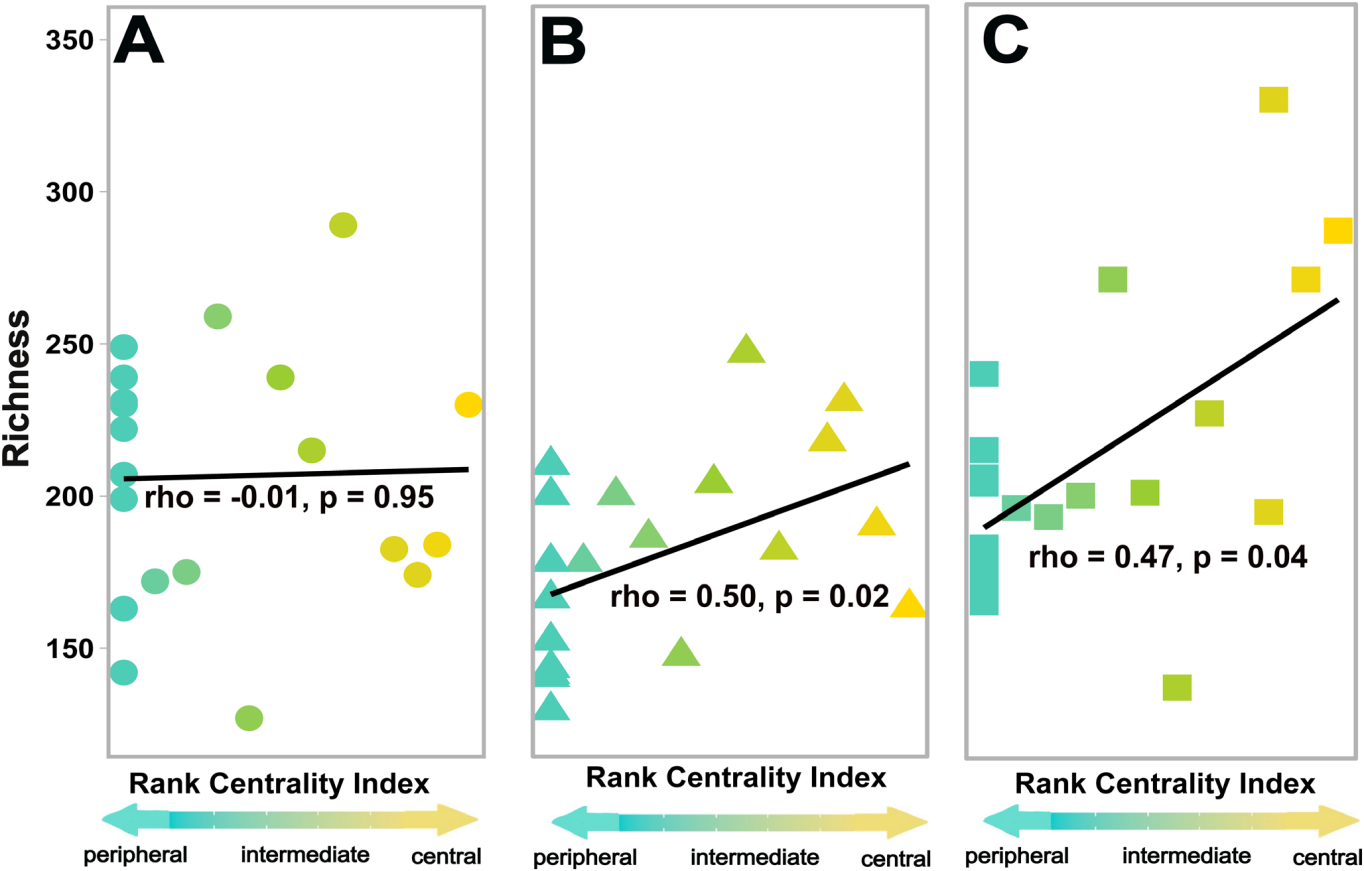
Central early colonising microbes significantly increased richness of early successional communities at later time points. Richness of assembling soil microbiomes at timepoints (A) 1 day post inoculation, (B) 7 days post inoculation and (C) 14 days post inoculation. Richness significantly increased with centrality of the experimentally inoculated early coloniser at the latter two time points. Richness axis values are shared for all panels. Statistics are given for Spearman correlations, and each point represents the richness response to one of the 20 experimental early colonisers used in the manipulative field experiment (calculated from pooled reads of the five replicate microcosms). Overlapping points indicate that multiple early colonisers shared the same richness responses.

**FIGURE 4:**
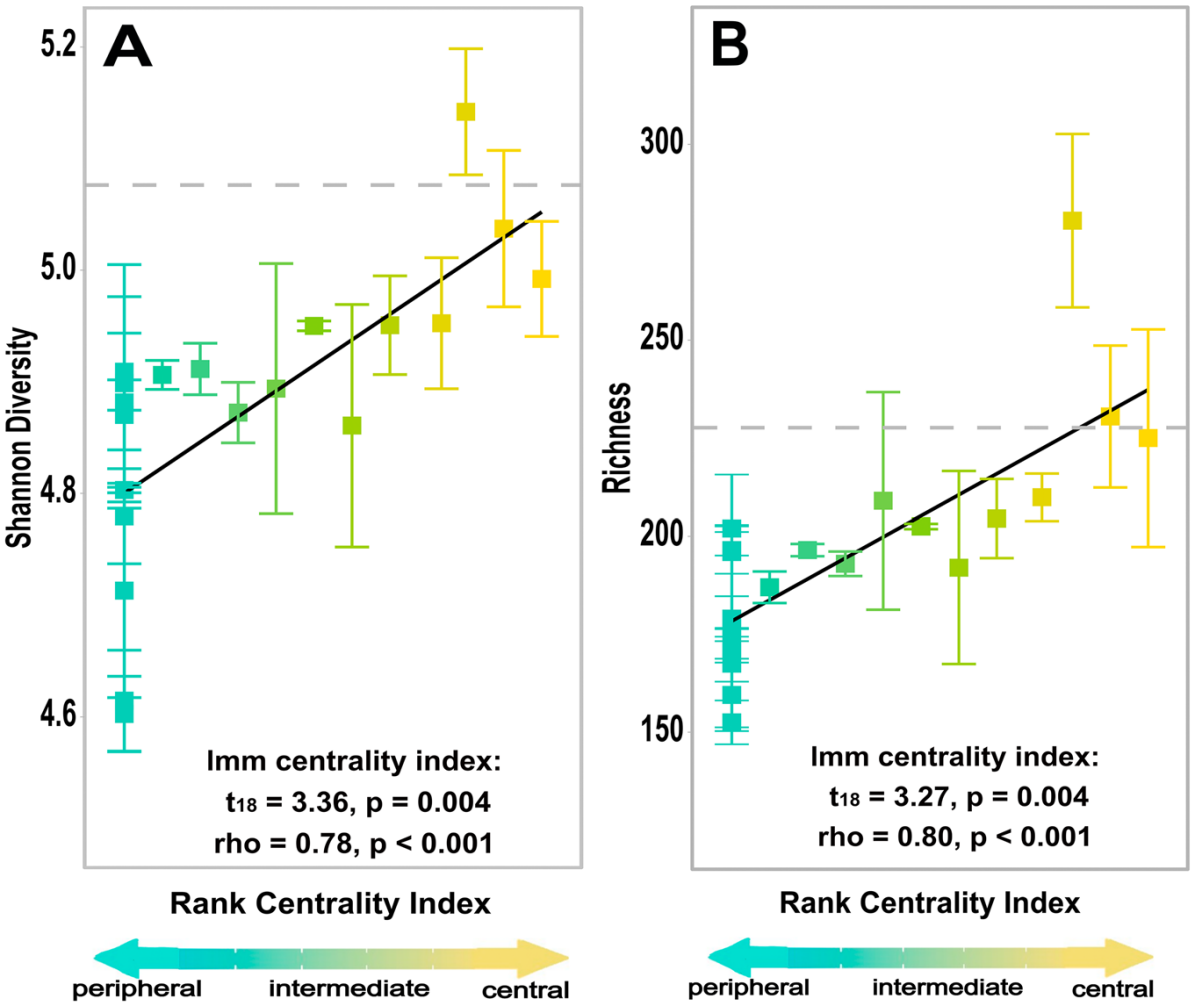
Central early colonising microbes significantly increased diversity and richness of early successional communities at later time points. Average (A) Shannon diversity and (B) richness of assembling soil microbiomes of timepoints 7 days post inoculation and 14 days post inoculation. Diversity and richness significantly increased with centrality of the experimentally inoculated early coloniser at the latter two time points. Statistics are given for the linear mixed model centrality index term as well as for Spearman correlations. Each bar depicts the standard error, and each point represents the average diversity or richness response to one of the 20 experimental early colonisers used in the manipulative field experiment (calculated from pooled reads of the 5 replicate microcosms). Grey dashed lines represent the diversity and richness for the natural community assembly (i.e., uninoculated controls).

We discovered that network connectivity is an important indicator of an early coloniser's role in supporting prokaryotic biodiversity as well as its impact on community composition and recruitment of other microbial taxa. For instance, centrality is a better predictor of diversity and richness than the other ecological attributes of landscape rarity and degree of local habitat specialisation (centrality index: *t*_18_ = 3.36, *p* = 0.004) (Figure S3). We found that at 1 day post inoculation, there were no differences in diversity (rho = 0, *p* = 0.99; Figure S5A) and richness (rho = −0.01, *p* = 0.95; Figure 3A) across microcosms. This is likely because not enough time had passed for microbial interactions to significantly impact community dynamics. However, by the latter two time periods, microcosms inoculated with high centrality early colonisers had assembled more diverse communities (rho = 0. 78, *p* < 0.001) with centrality of early colonisers alone explaining ~60% of the variance in diversity across microcosms (Figure 4A, Figure S5B,C). When breaking diversity into its components, we found that most of the differences in diversity could be attributed to differences in richness because centrality of early coloniser was a significant predictor of richness (*t*_18_ = 3.27, *p* = 0.004; Figures 3B,C and 4B) but not evenness (*t*_18_ = 0.288, *p* = 0.78; Figure S6). By the latest time period, central early colonisers had on average 35% richer communities than intermediate and 40% richer communities than peripheral groups (Figure 3C). The centrality of early colonisers explained on average ~ 64% of the total variance in richness of assembling communities (rho = 0.80, *p* < 0.001; Figure 4B).

**FIGURE 5:**
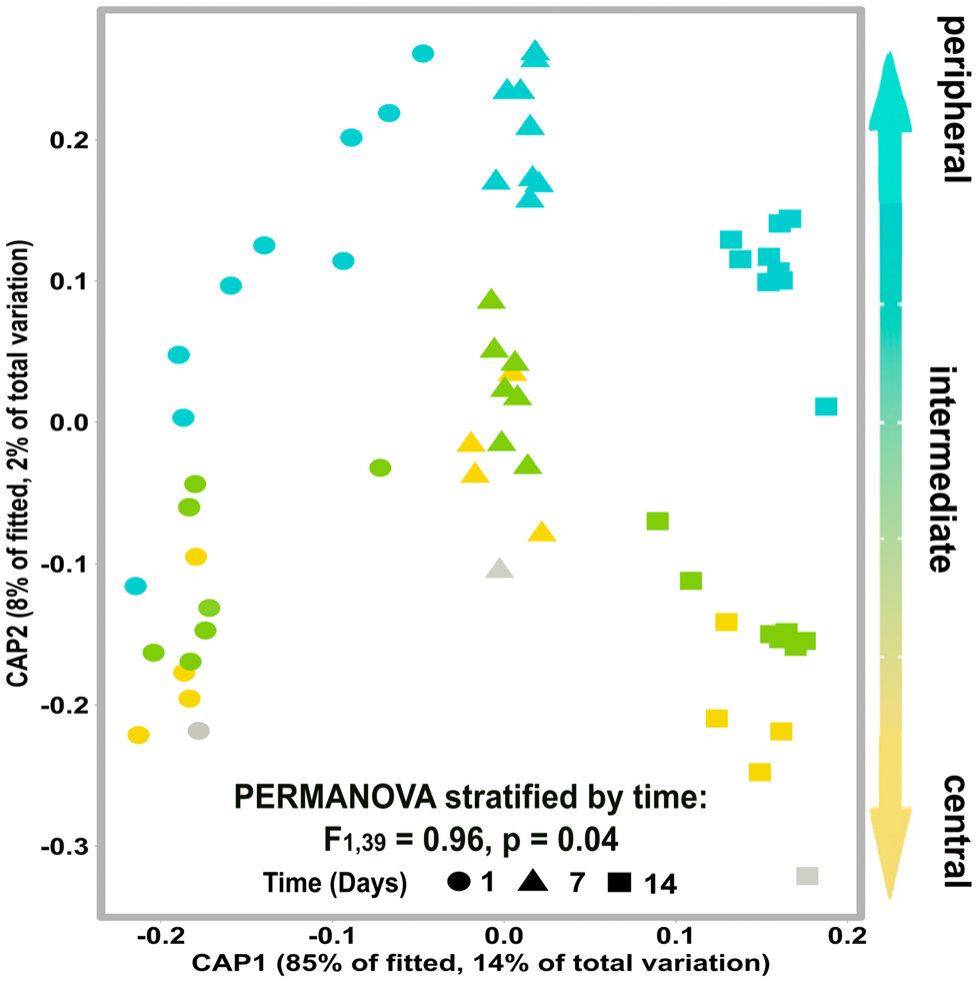
Different centralities of early colonising microbes lead to different community composition trajectories during early succession. Community composition was impacted by the centrality of early colonisers as can be visualised in this db‐RDA ordination of prokaryotic community composition, time, and centrality. Each point represents the prokaryotic community composition of a microcosm inoculated with different early colonisers. Points are coloured by early coloniser centrality and shapes represent the collection time (days post inoculation). Statistics are given for the PERMANOVA stratified by time using centrality of early colonisers as the explanatory variable. Grey points represent the community composition for the natural community assembly (i.e., uninoculated controls).

In a set of uninoculated microcosms, assembling communities had a mean diversity of 5.07 and a mean richness of 226 taxa, which is in the upper range of diversity and richness found in our experimental microcosms (Figure 4A,B). The assembling community composition from the uninoculated microcosms also clustered with that of the central microbes (Figure 5). Taken together, this suggests that natural early colonisers (i.e., those that establish through early dispersal) may be upper centrality microbes. When we evaluated the source pool for the assembling communities (i.e., the natural microbiomes from the surrounding rosemary scrub), we found a mean diversity of 5.07 and mean richness of 274 taxa, which again is similar to our most diverse and species‐rich experimental communities (found in the central microbe microcosms).

When we repeated diversity analyses using four prominently used alternative network construction methods (i.e., networks constructed using SparCC with ESV clustering, SpiecEasi MB, alternative seed, and rarefaction cross‐domain network normalisation), we found the same result that central early colonisers significantly increased diversity (ESV: *t*_17_ = 2.27, *p* = 0.04; SpiecEasi: *t*_18_ = 2.70, *p* = 0.01; seed: *t*_18_ = 2.78, *p* = 0.01; rarefaction: *t*_18_ = 2.21, *p* = 0.04; Figures S7, S8). This indicates that the importance of taxa's centrality in microbiome community assembly is robust to many different network construction decisions. Overall, our results demonstrate that highly central microbes can act as keystone taxa during early succession by increasing prokaryotic community‐wide diversity, which has been linked to greater microbiome stability, resistance to perturbations and soil functional capacity (Xun et al. 2021).

### 3.3 Centrality of Early Colonising Microbe Predicts Prokaryotic Community Composition

The keystone effects of central microbes were also illustrated by changes in prokaryotic microbiome composition with differences in centrality of early colonisers impacting community‐wide structure (PERMANOVA with time as strata; *F*_1,39_ = 0.96, *p* = 0.04; Figure 5). Despite replicates within each centrality tier being biologically unique (i.e., different early coloniser taxa), the assembled communities clustered by the coloniser's centrality. Importantly, this indicates that central microbes predictably create more similar communities regardless of their taxonomic identity. While patterns of succession observed across diverse microbial communities have often been attributed to habitat filtering (Ortiz‐Álvarez et al. 2018), our results suggest that connectivity of the early coloniser also plays a significant role in the predictability and trajectory of prokaryotic succession.

### 3.4 Central Taxa Disproportionately Recruit Other Influential, Highly Connected Taxa into Assembling Prokaryotic Communities While Peripheral Taxa are Minimally Affected By Other Microbes

**FIGURE 6:**
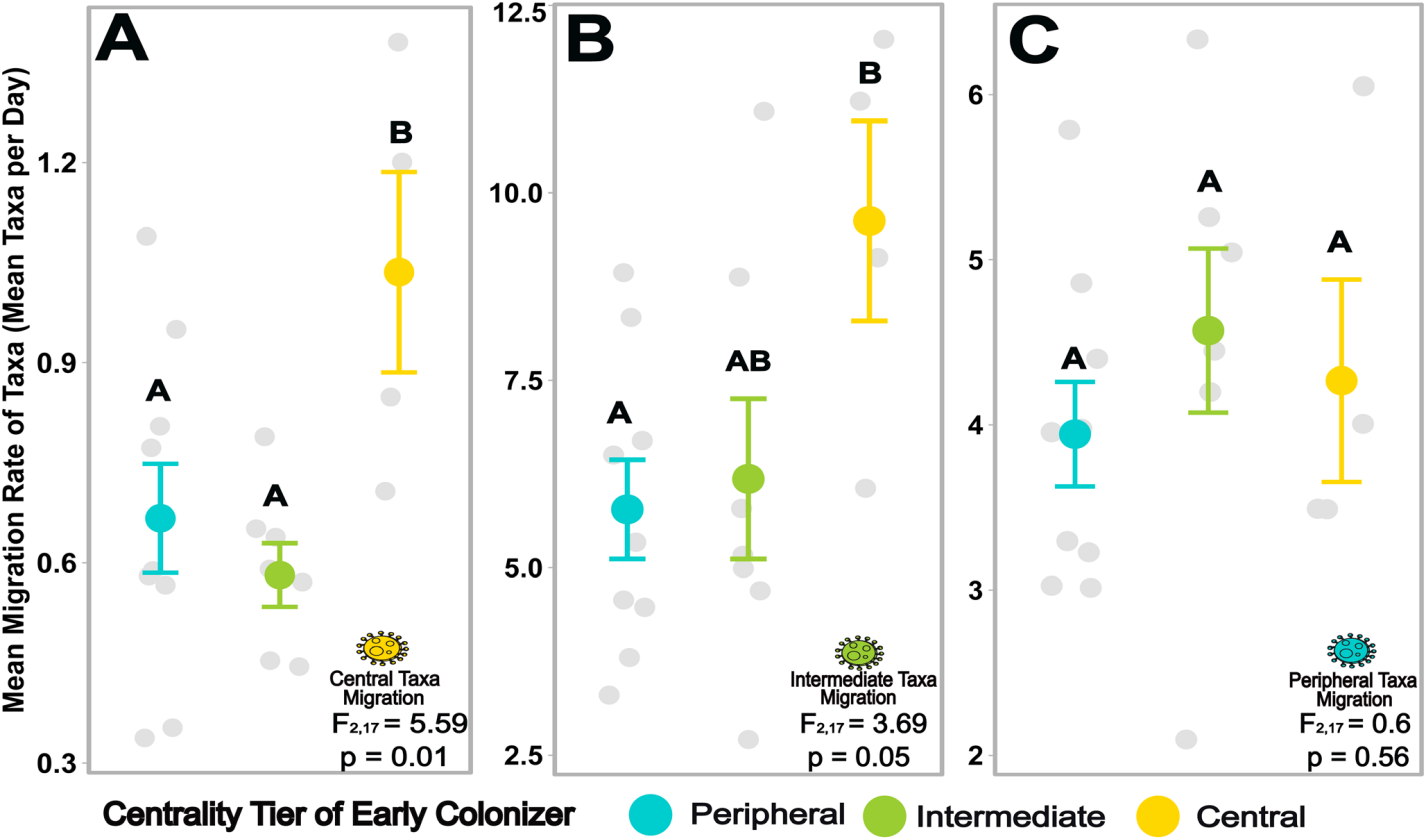
Communities with central early colonisers have higher recruitment of other structurally important, central taxa while recruitment of peripheral, transient microbes is unchanged. Centrality of early colonisers also affected the structure of the microbiome with central early colonisers recruiting significantly more central and marginally more intermediate microbes to their communities while peripheral recruitment did not differ. The mean migration rates (number of taxa in assembled community divided by total days) of the assembling central (A), intermediate (B), and peripheral (C) taxa are depicted across the microbiomes for each centrality tier of the early coloniser. Grey points are the values for each of the 20 experimental early colonisers (*n* = 9 peripheral inoculant group, *n* = 7 intermediate inoculant group, *n* = 4 central inoculant group). Coloured points depict the means for each early coloniser centrality tier with standard error bars. Different letters denote significant differences (*p* < 0.05) from an ANOVA followed by a post hoc Tukey's test.

In addition to enhancing diversity and influencing community make‐up, central early colonisers altered community structure by increasing recruitment of other central microbes. We compared how central, intermediate and peripheral early colonisers impacted recruitment of other microbes in each of these categories during early succession. Interestingly, central early colonisers recruited 66% more central taxa to their newly assembling communities per day than less‐connected early colonisers (ANOVA; *F*_2,17_ = 4.7, *p* = 0.02) (Figure 6A), indicating that these central microbe structure communities by disproportionately recruiting other potentially influential microbes. These effects on the nonrandom recruitment of other taxa are further demonstrated by the marginal increase in migration of intermediate microbes into microcosms with central early colonisers compared with intermediate and peripheral early colonisers (ANOVA; *F*_2,17_ = 3.8, *p* = 0.04) (Figure 6B). In contrast, the migration of peripheral taxa was independent of early coloniser centrality (ANOVA; *F*_2,17_ = 0.60, *p* = 0.60) (Figure 6C), validating that peripheral taxa are acting as stochastic, transient microbes minimally affected by other microbes. Overall, these findings demonstrate that the increase in diversity in communities with central early colonisers results from the recruitment of structurally important taxa rather than transient, peripheral taxa.

## 4 Discussion

Soil microbiomes impact all natural and agro‐ecosystems by regulating the terrestrial global stock of carbon, nitrogen and critical nutrients, which regulate primary productivity (Sokol et al. 2022). Due to the escalating stresses of the Anthropocene, these microbes have become even more essential as they are able to bioremediate pollution (Abatenh et al. 2017), promote restoration of degraded habitats (Valliere et al. 2020) and confer plant drought and salinity tolerance (Ma et al. 2020). Despite the facts that soil microbiome's harbour a quarter of earth's biodiversity (Guerra et al. 2021) and affect the health of all organisms on the planet including humans (Banerjee and van der Heijden 2023), research on understanding healthy soil formation remains underrepresented (Banerjee and van der Heijden 2023). In order to preserve this biodiversity and the ecosystem services it provides, we need to be able to identify the keystone taxa that disproportionately promote diversity and structure within their natural soil communities. Our study contributes to this goal by combining culturing, sequencing, network theory and manipulative field experiments to demonstrate for the first time that centrality is a powerful indicator of which microbial taxa are keystone species for community assembly in nature. Importantly, we find, that central early colonisers: (1) increased biodiversity, (2) structured communities and (3) deterministically recruited a more connected prokaryotic community during early succession. This indicates that central placement of taxa within community networks can be used to successfully identify structurally significant microbes in complex communities, which opens new opportunities for effectively engineering and restoring microbiomes through intermicrobial interactions. Below, we discuss how our results elucidate multiple important principles of soil microbiome assembly and new directions for environmental microbiome research sparked by the discovery that central microbes are keystones.

Our study highlighted three important principles of soil microbiome assembly. First, we found evidence that deterministic community assembly depended on the connectedness of the early coloniser microbes. Studies of biocrusts across a multitude of habitats from subtropical forests (Liu et al. 2021), grasslands (Albright et al. 2019), deserts (Xu et al. 2020; Zhou et al. 2024) and even potash salt heaps (Ohan et al. 2023) have hypothesised based on observational evidence that deterministic processes rather than stochastic processes are the dominant forces in early prokaryotic community assembly. These same studies also suggest that there is limited diversity in early fungal establishment and that fungal arrival is stochastic during early assembly but shifts to becoming deterministic by late succession. Because we were interested in early successional communities, we chose to focus on prokaryotic assembly and our results provide the first experimental support from nature to substantiate this hypothesis that deterministic processes shape early prokaryotic assembly and demonstrate that connectivity of a microbe (prokaryote or fungi) to other microbes within networks is a prominent signal of a microbe's influence on early successional prokaryotic communities.

Second, keystone microbes appear to recruit other influential microbes within the soil communities. Specifically, we discovered that central early colonisers increased biodiversity through recruitment of other central taxa rather than stochastically recruiting a broader array of random, transient microbes. Given that centrality was a strong predictor of keystone roles in our experiment, the fact that migration of other central taxa into assembling communities almost doubled in communities initiated with central taxa supports a novel and surprising conclusion that keystone microbes selectively facilitate other likely keystone microbes. Third, peripheral taxa are ‘transient’ microbes that are unaffected by interactions with the keystone species or the make‐up of the assembling community. Unlike central microbes (and to some extent intermediate microbes), migration of peripheral microbes into assembling communities did not depend on the early coloniser nor the compositional differences they caused in the assembling communities, suggesting interactions with the microbial community are not as important for determining peripheral taxa's distributions. This makes sense if co‐occurrence links in networks represent species interactions (at least some of the time), then unlinked taxa would be expected to be less affected by interactions with other microbes. Recently, simulations of large microbiome data sets have led to researchers to postulate that consistency of microbes across samples (i.e., consistency in ‘occupancy’) can distinguish between transient and core components of microbiomes, with occupancy of relatively few samples indicating a transient microbe (Custer et al. 2023). Our study provides empirical evidence supporting this hypothesis since the peripheral microbes in our study were those taxa that occupied less than 10% of sites across the landscape. Our result that the peripheral taxa recruited at equal rates across all microcosm communities provides some of the first experimental validation in nature that these microbes are indeed acting as transient microbes that are minimally affected by other microbes. Together these three findings, provide some important insight into microbiome community dynamics by showing that central microbes are keystone taxa that can drive deterministic changes in their communities that these changes occur—at least in part—by facilitating other central microbes, and that low occupancy, peripheral taxa are stochastic elements of communities that are seemingly less impacted by microbe–microbe interactions.

Intriguingly, we also found that central, keystone microbes have both stronger positive and negative associations within networks than less‐connected groups, suggesting that keystones may promote increased diversity utilising a combination of positive and negative interactions. We hypothesise that microbe‐mediated environmental filtering may be occurring where the central taxa change the soil conditions in a manner that increases the establishment of a wide diversity of microbes. Likely positive mechanisms underlying keystone‐enhanced diversity include cross‐feeding/metabolite exchange with other microbes (Hoek and Merks 2017) and reduction in environmental stress via formation of a structural barrier (Yin et al. 2019), while a likely negative mechanism is the ‘keystone predator’, whereby a microbe prevents dominating competitors from establishing/spreading (Ghoul and Mitri 2016). To determine the relative importance of these mechanisms, we advocate for future research focussed on characterising the functional roles that these keystone microbes as well as other hub taxa play within their respective communities. For instance, with the declining cost of sequencing, metagenomics could be used in future to functionally profile both central and less‐connected microbes within communities by evaluating functional genetic variation within Metagenome‐Assembled Genomes (MAGs) of these two groups. Further, lower cost, better quality whole genome sequencing of culture collections (Tyler et al. 2018) in conjunction with recent improvements in metatranscriptomics (Westermann and Vogel 2021) could be leveraged in future work to determine which functions keystone microbes are performing during different stages of succession. Knowledge of keystone functional roles coupled with our validated framework for identifying keystone microbes could provide a new avenue for more effective monitoring of microbiome health in natural environments.

Our findings also open up other important avenues for future research that will inform our understanding of keystone species and the predictive value of microbial network properties. First, future work evaluating how much redundancy exists for keystone functions is crucial for understanding the resilience of microbial communities to disturbance and the escalating stress of the Anthropocene. Recent studies found the multifunctionality of soil microbiomes was most strongly disrupted when microbiomes experienced multiple stressors simultaneously, well beyond what would be expected based on the effects of each stressor separately (Rillig et al. 2019; Rillig et al. 2023), suggesting functional redundancy is likely important within microbiomes. Therefore, understanding whether and when redundancy exists for keystone roles and functions within these communities could provide important insight into predicting ecosystem functional stability. Second, another area for future exploration is understanding the consistency of microbial roles within networks across time and space. Previous studies of plant‐associated microbiomes have found evidence for conservation of community and network structure across years (within a season), and some network properties, such as hub taxa, can predictively change between seasons within a year (Almario et al. 2022; Shi et al. 2016; Zhang et al. 2020). Our study, which focussed on hub taxa identified in the same season as they were tested for keystone roles, found that the network property of centrality was able to predict how these microbes impacted community assembly. Future work evaluating when meaningful changes in time (such as seasonal variation) or in space (such as local variation in environmental stress) lead to changes in keystone roles and centralities of microbes would be especially valuable for progressing the predictive use of networks and how keystones function within microbial communities.

A major goal of environmental microbiology is to utilise microbial inoculations to promote ecological diversity and resilience in sustainable agriculture and habitat restoration, similar to what is currently being done with reintroduction of macro‐organisms. Successful reintroductions of keystone macro‐organisms including those acting as top predators (grey wolves, Ripple and Beschta 2012), large herbivores (bison, McMillan, Kunkel, Hagan, and Jachowski, 2019; antelope, Guyton et al. 2020) and ecosystem engineers (prairie dogs, Davidson et al. 2018; beavers, Hooker et al. 2024) into their historical habitats have generated levels of diversity and richness similar to that of an undisturbed habitat. We found analogous results where inoculation with a hub microbe facilitated a high level of microbial diversity and richness similar to natural controls, further strengthening our conclusion that these hubs are acting as keystones, which could provide a new avenue for environmental engineering of microbiomes. We know from the extensive work done on animal microbiomes that foundational knowledge in host community assembly and microbial function are crucial for the development of practical applications. This information has been used to successfully treat gastrointestinal diseases of humans and promote gut health of feed animals to reduce antibiotic use (Foo et al. 2017). However, the knowledge of environmental microbiomes still lags behind host‐associated microbiomes, and more research is needed if we want to conserve microbial diversity and successfully harness environmental microbiomes for globally important processes such as nutrient cycling, climate regulation and land degradation restoration (Silverstein et al. 2023). We show that using network theory with data from natural communities has promising potential for the discovery of key microbial players across unexplored habitats and that future functional studies could help to unearth additional pathways for microbial engineering.

## Author Contributions

All authors contributed to the conceptualisation and design of the study. A.H.R. and D.J.H. isolated and characterised the culture collection. A.H.R. set up, managed, collected and processed all samples from the manipulative field experiment microcosms. A.H.R. performed the data analyses with assistance from D.J.H. for the network analyses. M.E.A. acquired the necessary funding for the projects and mentored participating graduate students. All authors wrote and contributed to the editing of the manuscript.

## Conflicts of Interest

The authors declare no conflicts of interest.

### Peer Review

The peer review history for this article is available at https://www.webofscience.com/api/gateway/wos/peer‐review/10.1111/ele.70031.

## Supporting information


Data S1.

File S1.

File S2.

File S3.

File S4.

## Data Availability

Raw sequence data is available at NCBI GenBank (PRJNA1028569), package versions for scripts are available in File S4, and comprehensive supporting data and scripts are available at Zenodo (https://doi.org/10.5281/zenodo.14056956), version v5 (Rawstern, Hernandez, and Afkhami 2024).
