# Supplementary material for: Central Taxa Are Keystone Microbes During Early Succession
Source: Ecol Lett. 2024 Dec 31;28(1):e70031. doi: 10.1111/ele.70031 (PMC11687413; doi:10.1111/ele.70031)
Supplement: Supplementary file 1 — Data S1. [file ELE-28-0-s002.docx]

**Supplementary Methods:**

***Evaluating robustness of network construction***

To evaluate the robustness of our conclusions about the role of centrality in microbial community assembly from our field experiment, we also conducted several follow up analyses related to network construction methods. We constructed networks in 4 additional ways with different 1.) clustering methods (Exact Sequence Variants (ESVs)), 2.) algorithms (*denovo* 97% clustering network using SpiecEasi Meinshausen-Buhlmann's neighborhood selection (MB)), 3.) seeds (repeat of *denovo* 97% clustering network with another seed of randomness), and 4.) normalization methods (cross-domain network normalized using rarefaction). Networks were constructed with default parameters in Fastspar (v1.0.0; Watts et al. 2019) except for the network which used SpiecEasi (v1.1.1; Kurtz et al. 2015). We recalculated centrality indices for each network and confirmed in all cases that using a different network construction method led to the same qualitative results/conclusions.

***Isolation and identification of rosemary scrub microbial taxa***

We isolated microbial taxa from the Florida Rosemary Scrub using soils (Hernandez et al. 2021) collected across Archbold Biological Station (Fig. 1A). We suspended 1 gram of soil in 50 mL of sterile water, serially diluted to a final concentration of 10-5 µg/µL and plated 200 µL of solution. We used 48 combinations of yeast extract agar plates with 4 carbon sources (mannitol, glucose, sucrose, maltose), 3 pH levels (6, 7, 8), and 4 incubation temperatures (23°C, 28°C, 37°C, 55°C) (Fig. 1B). Sugars were filter-sterilized at a 10% concentration and added after the media was autoclaved to prevent oxidation. Isolated taxa were cataloged for unique morphological characteristics, purified in liquid media, preserved as glycerol stocks (Estrada-Navarrete et al. 2007), and stored at -80°C.

To determine the isolated taxa’s identity, we extracted DNA for Sanger sequencing using the E.Z.N.A. Soil DNA Kit following the manufacturer’s protocol (OMEGA Bio-Tek, Norcross, GA). Four negative controls were also included, in which 500 µL of sterile water was used in place of a microbial pellet to test for contamination during the extraction process. DNA quantity was checked using a Qubit 4 fluorometer (Qiagen, Carlsbad, CA) and no cross-contamination occurred. Ribosomal DNA was targeted using primers 515F and ITS7 for PCR. We purified the genomic DNA using an E.Z.N.A Gel Extraction Kit and combined 15 µL of purified DNA with 2 µL 515F (10 µM) for prokaryotic samples or 2 µL ITS7 (10 µM) for fungal samples. DNA was Sanger-sequenced at Eurofin Genomics (Louisville, KY, USA).

***Set-up of experimental microcosms***

To create experimental microcosms, sterile pots were filled with 500 g of sandy soil from the biological soil crust of the Florida Rosemary Scrub at Archbold Biological Station (same site as the field experiment) that was sterilized at 121°C three times (at least 24 hours between each sterilization). Under aseptic conditions, microcosms were inoculated with 5 mL of microbial inoculum (1 x 10^6^ cells per mL sterile water) from one of the 20 isolated taxa or with 5 mL of sterile water as a control (21 treatments x 5 replicate microcosms = 105 total microcosms)(Fig. 1D). The inoculated microcosms ultimately contained 1 x 10^4^ cells per g of soil, which is approximately 1% of the microbial abundance in typical rosemary scrub soil samples as determined by qPCR of our broader community sequencing across 64 rosemary scrub habitat patches (Hernandez et al. 2021). This inoculum amount represents biologically relevant levels of abundance for an individual early colonizer species in a natural habitat after a severe fire (Ammitzboll et al. 2021). All pots received an additional 15 mL of sterile water to distribute the inoculum throughout the soil and prevent desiccation during experiment set up. The microcosms were buried 1 cm into the field soil so that the openings on the bottoms of microcosms were in contact with the soil surface for potential microbial migration from below as well as from the air (Carrington 2010). These openings also prevented the microcosms from becoming unnaturally water-logged during rain.

***Microbiome DNA extraction and sequencing***

DNA was extracted from each soil sample (n = 315; 21 treatment groups, 3 time points, 5 replicates) using the DNeasy PowerSoil Pro QIAcube HT Kit following modified protocols detailed elsewhere (Revillini et al. 2022). All negative controls had undetected fluorometer readings indicating contaminants were not present during the extraction process. Prokaryotic (archaea/bacteria) DNA was targeted using primer pairs 515F/806R for PCR (Caporaso et al. 2012). Libraries were prepared for sequencing using a two-step dual indexing protocol (Gohl 2016). After each PCR step, magnetic bead cleaning was performed and DNA quality was checked using 1% agarose gel electrophoresis. Indexed DNA from all 315 field experiment samples were pooled in equimolar quantities. Libraries were sequenced on an Illumina MiSeq Sequencer (v3, 300 bp paired end) at the University of Miami Center for Genome Technology (Miami, FL, USA). Sequencing primers (Revillini et al. 2022) were used that matched the universal tail sequences from the first round of amplification.

Sequences were processed through QIIME2 pipeline (Bolyen et al. 2019) (v.2022.2) to join paired-end reads and remove low quality bases. Denoising was performed with the DADA2 algorithm (Callahan et al. 2016), which removed chimeric sequences and truncated amplicon forward and reverse reads to an equal length generating exact sequence variants (ESVs). All ESV abundance tables from QIIME2 were imported into R v4.3.0 (R Core Team 2023). Replicates were pooled and rarefied (to 6000 reads) based on rarefaction curve analysis (R package GUniFrac, v1.7; Chen et al. 2012). Following best practices (Reitmeier et al. 2021), sequences with relative abundances of less than 0.25% were removed to avoid overinflating diversity due to spurious sequences.

**Supplementary Figures:**

**
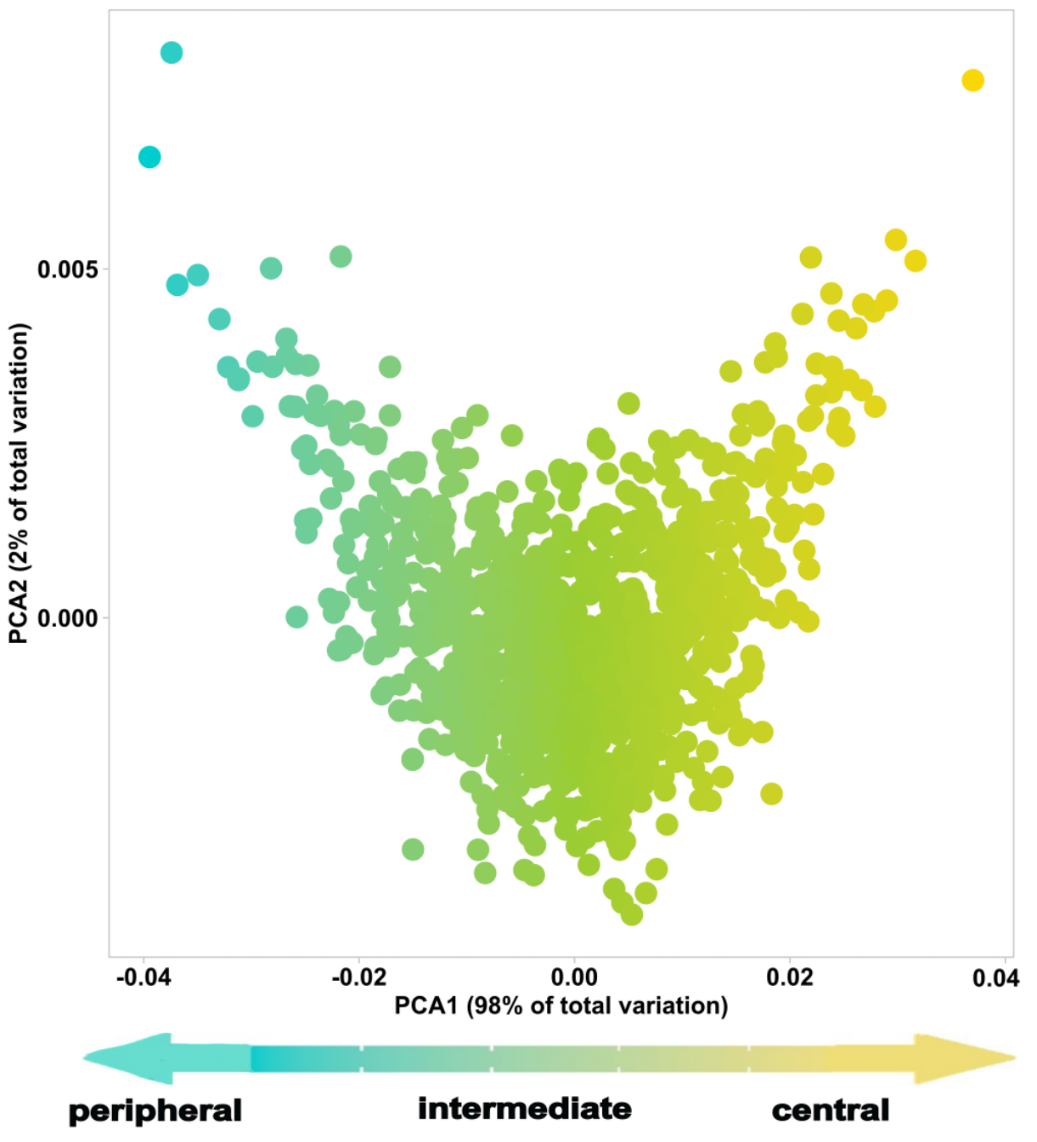
**

**Supplementary Fig 1. Common centrality metrics were combined into a composite index.** Degree centrality (the number of edges a node has connecting it to other nodes in the network), betweenness centrality (number of shortest paths between pairs of nodes that uses the node), closeness centrality (inverse distance to other nodes), and eigen centrality (transitive influence of node) values were used in a principal components analysis to calculate a composite index centrality. The composite index centrality is PC value from the primary axis of variation (i.e. axis that explained ~98% of variation). Taxa were binned into centrality tiers using the following criteria: the top 5% of all microbes with the highest centrality index (centrality index range: 0.037 to 0.007) were classified as central (predicted keystones), the other microbes in the top 20% of centrality index were classified as intermediate (centrality index range: 0.007 to -0.04), and the remaining microbes (bottom 80% of centrality index) were considered peripheral microbes (i.e. transient). Peripheral microbes were assigned the minimum centrality index (-0.04) since these taxa fall outside of the network.


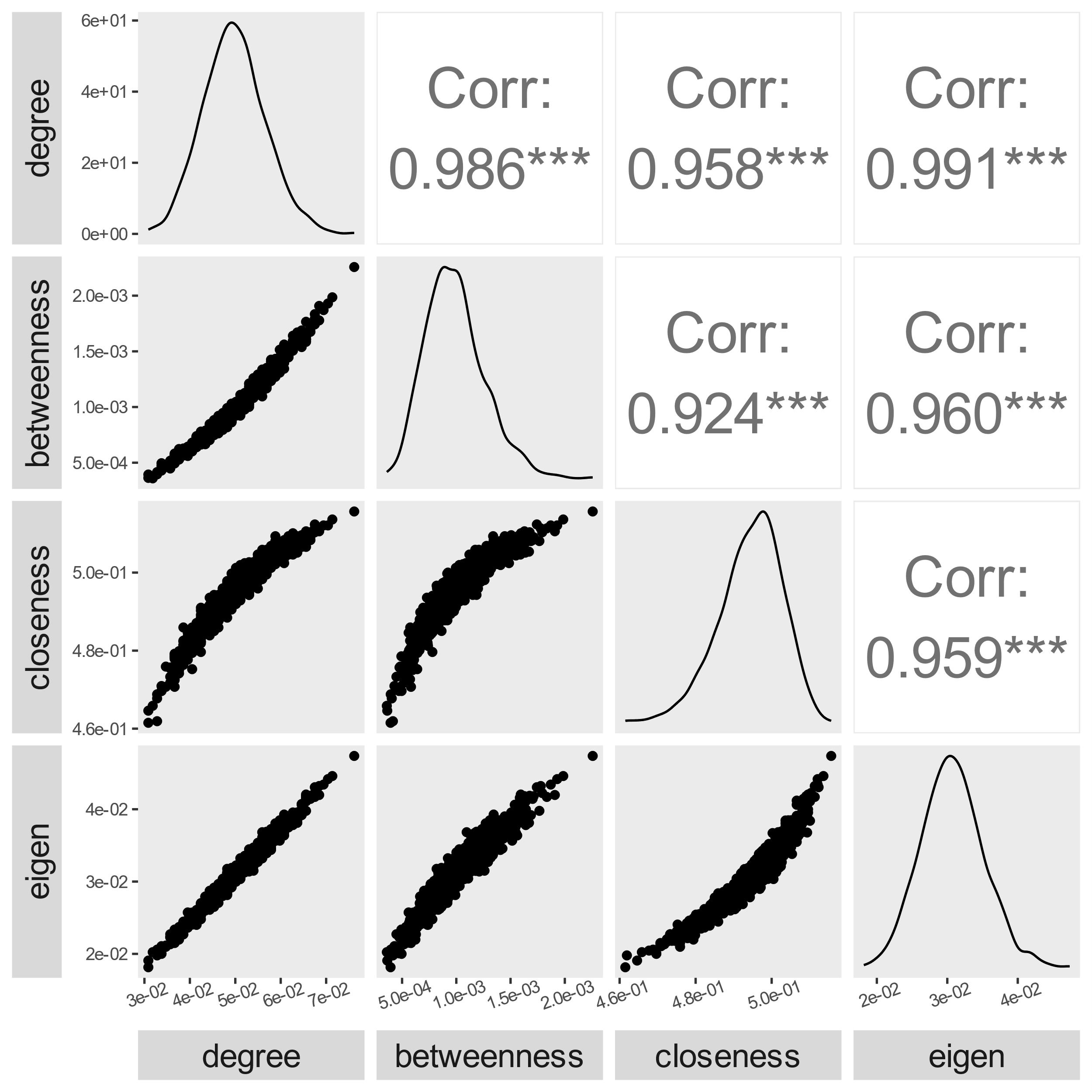


**Supplementary Fig 2. All network centrality metrics were all highly correlated for microbial nodes.** Degree centrality (the number of edges a node has connecting it to other nodes in the network) is highly correlated with betweenness centrality (number of shortest paths between pairs of nodes that uses the node), closeness centrality (inverse distance to other nodes), and eigen centrality (transitive influence of node). Statistics and correlation plots are given for the Pearson correlation coefficient (r) value between pairs of centrality variables and the three asterisks denote that the p values are <0.001. Histograms represent the distribution of all nodes across the network.


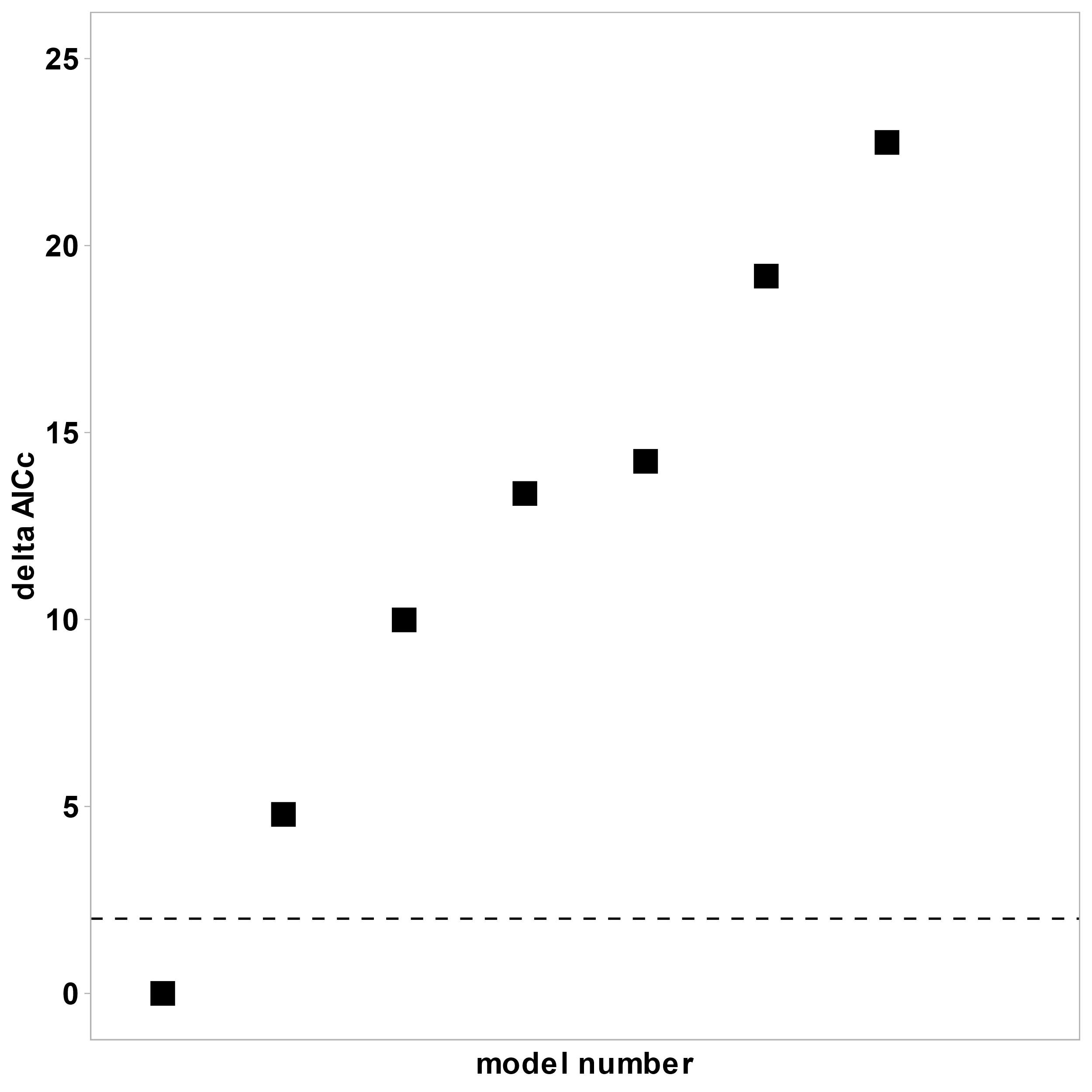


**Supplementary Fig 3. Global model selection results for the diversity of assembling microbiome in our experiment.** The fixed effects terms considered in the full model were landscape rarity, specialization index, and centrality index of the early colonizing microbe the microcosm received, and the random effect was time point of microbiome collection within treatment group. Only the best model (Shannon diversity ~ centrality of early colonizer, random = ~1|treatment) had a delta AICc under the threshold of 2 (represented by dashed black line).


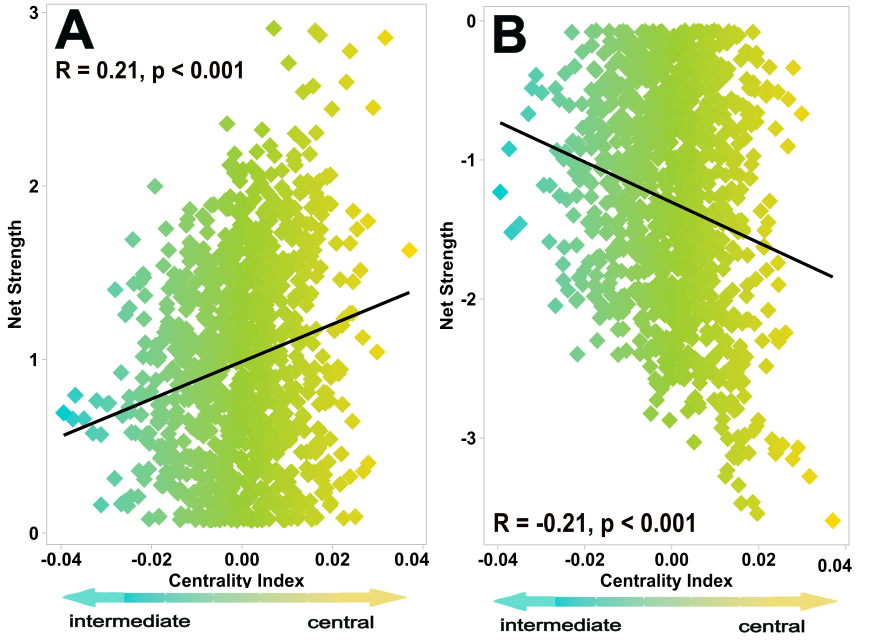


**Supplementary Fig 4. Central microbes show higher net positive and net negative strength than intermediate taxa.** **(A)** Net positive strength (number of positive edges x average edge correlation coefficient) and **(B)** net negative strength (number of positive edges x average edge correlation coefficient) significantly increased with centrality index across all network nodes. Statistics are given for Pearson correlations.


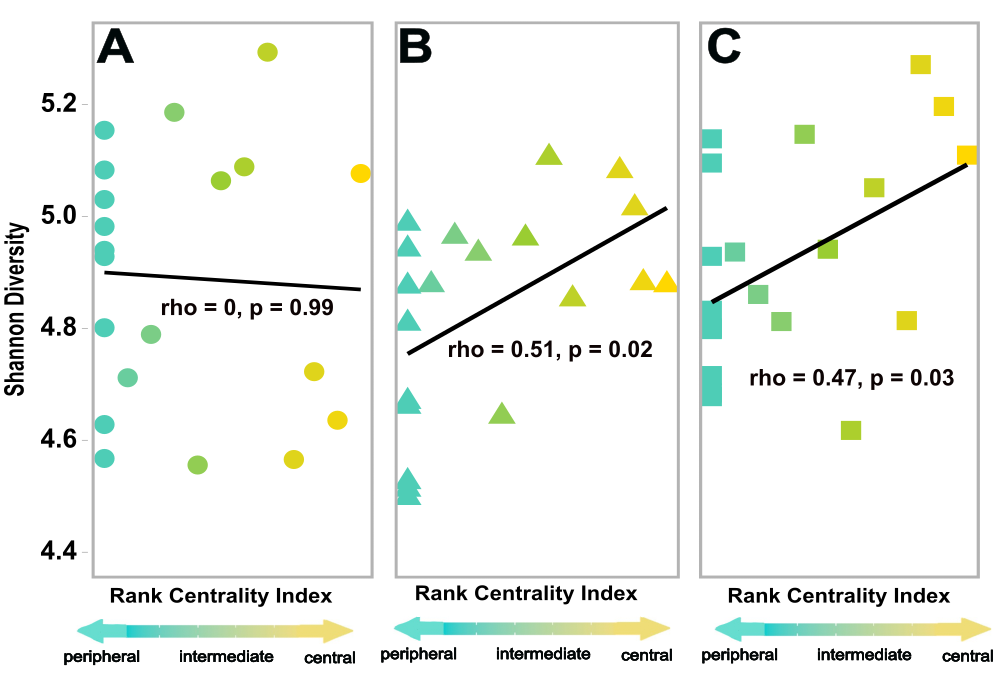


**Supplementary Fig 5. Central early colonizing microbes significantly increased diversity of early successional communities at later time points.** Shannon diversity of assembling soil microbiomes at timepoints **(A)** 1-day post-inoculation,  **(B)** 7-days post-inoculation, and **(C)** 14-days post-inoculation.  Diversity significantly increased with centrality of the experimentally inoculated early colonizer at the latter two time points. Shannon diversity axis values are shared for all panels. Statistics are given for Spearman correlations, and each point represents diversity response to one of the 20 experimental early colonizers used in the manipulative field experiment (calculated from pooled reads of the 5 replicate microcosms).


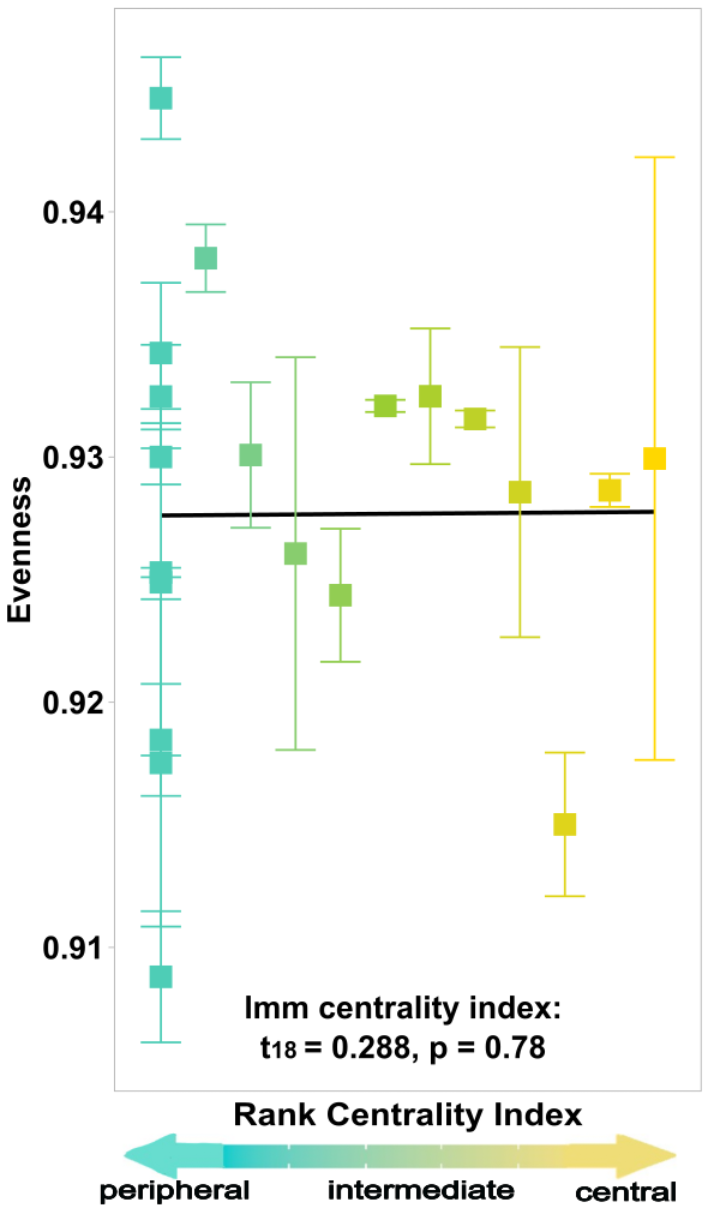


**Supplementary Fig. 6. All centrality types of early colonizing microbes have the same evenness in their successional communities at later time points.** Average evenness of assembling soil microbiomes of timepoints 7-days post-inoculation and 14-days post-inoculation. Statistics are given for the linear mixed model centrality index term. Each bar depicts the standard error and each point represents the average evenness response to one of the 20 experimental early colonizers used in the manipulative field experiment (calculated from pooled reads of the 5 replicate microcosms).


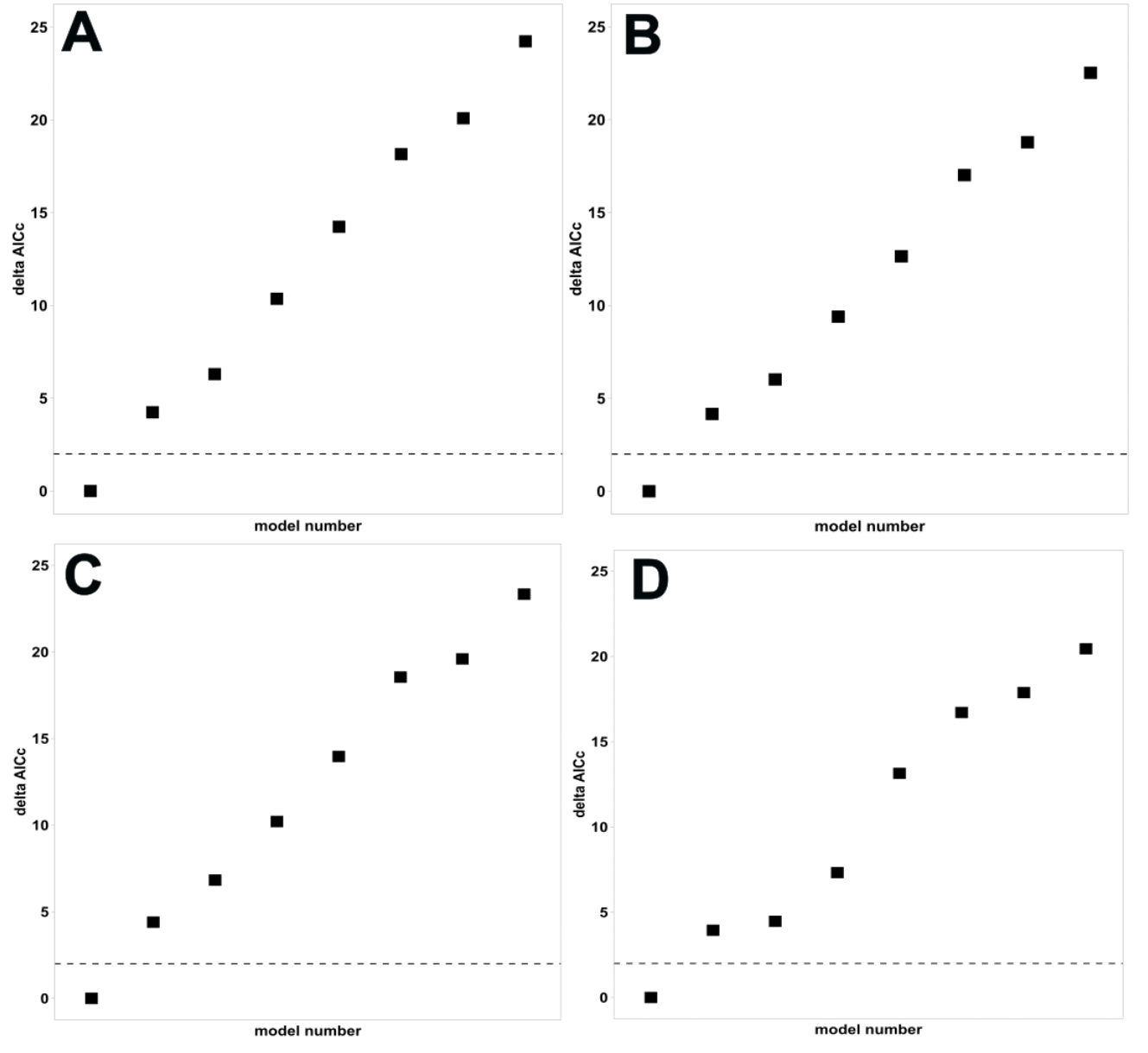


**Supplementary Fig 7. Global model selection results for the diversity of assembling microbiome is the same for alternative network construction methods.** The fixed effects terms considered in the full model were landscape rarity, specialization index, and centrality index of the early colonizing microbe the microcosm received, and the random effect was time point of microbiome collection within treatment group. Only the best model (Shannon diversity ~ centrality of early colonizer, random = ~1|treatment) had a delta AICc under the threshold of 2 (represented by dashed black line) for all additional network construction types of **(A)** SparCC ESV clustering, **(B)** SpiecEasi MB 97% denovo clustering, **(C)** SparCC 97% denovo clustering with different seed, and **(D)** SparCC 97% denovo clustering normalization with rarefaction.


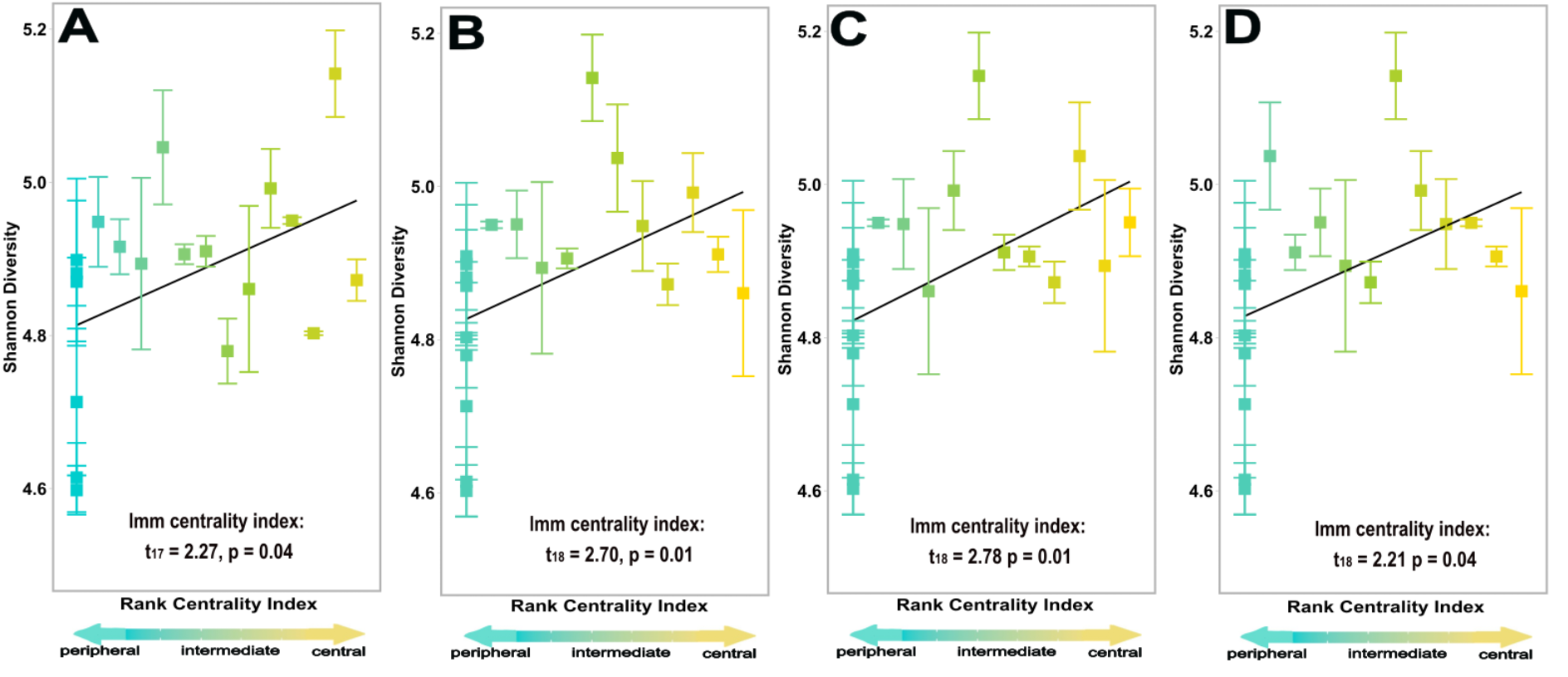


**Supplementary Fig 8. Central early colonizing microbes significantly increased diversity of early successional communities at later time points using additional different common network construction methods.** Average Shannon diversity of assembling soil microbiomes of timepoints 7-days post-inoculation and 14-days post-inoculation using networks constructed from **(A)** SparCC ESV clustering, **(B)** SpiecEasi MB 97% denovo clustering, **(C)** SparCC 97% denovo clustering with different seed, and **(D)** SparCC 97% denovo clustering normalization with rarefaction. Diversity significantly increased with centrality index of the experimentally inoculated early colonizer at the latter two time points. Statistics are given for the linear mixed model index centrality term which was the best model based on model selection with lowest delta AIC_c_ in all cases . Each bar depicts the standard error and each point represents the average diversity response to one of the 20 experimental early colonizers used in the manipulative field experiment (calculated from pooled reads of the 5 replicate microcosms).

**Supplementary References:**

Ammitzboll, H., Jordan, G., Baker, S., Freeman, J., Bissett, A. (2021). Diversity and abundance of soil microbial communities decline, and community compositions change with severity of post-logging fire. *Mol. Ecol.* **10,** 2434-2448.

Bolyen, E., Rideout, J., Dillon, M., Bokulich, N., Abnet, C., Al-Ghalith, G. *et al.* (2019). Reproducible, interactive, scalable, and extensible microbiome data science using QIIME 2. *Nat. Biotechnol.* **34,** 852–857.

Callahan, B., McMurdie, P., Rosen, M., Han, A., Johnson, A., Holmes, S. (2016). DADA2: High-resolution sample inference from Illumina amplicon data. *Nat. Methods.* **13,** 581-583.

Carrington, M. (2010). Effects of soil temperature during fire on seed survival in Florida Sand Pine Scrub. *Int. J. For. Res.* **2010,** 402346.

Chen, J., Bittinger, K., Charlson, E., Hoffmann, C., Lewis, J., Wu, G. *et al.* (2012). Associating microbiome composition with environmental covariates using generalized UniFrac distances. *Bioinform.* **28,** 2106-2113.

Caporaso, J., Lauber, C., Walters, W., Berg-Lyons, D., Huntley, J., Fierer, N. *et al.* (2012). Ultra-high-throughput microbial community analysis on the Illumina HiSeq and MiSeq platforms. *ISME J.* **6,** 1621-1624.

Estrada-Navarrete, G., Alvarado-Affantranger X., Olivares, J., Guillén, G., Díaz-Camino, C., Campos, F. *et al.* (2007). Efficient and reproducible genetic transformation of Phaseolus spp. by *Agrobacterium rhizogenes*. *Nat. Protoc.* **2,** 1819-1824.

Gohl, D., Vangay, P., Garbe, J., MacLean, A., Hauge, A., Becker, A. *et al.* (2016). Systematic improvement of amplicon marker gene methods for increased accuracy in microbiome studies. *Nat. Biotechnol.* **34,** 942–949.

Hernandez, D., David, A., Menges, E., Searcy, C., Afkhami, M. (2021). Environmental stress destabilizes microbial networks. *ISME J.* **15,** 1722-1734.

Kurtz, Z., Müller C., Miraldi E., Littman D., Blaser M., Bonneau, R. (2015). Sparse and compositionally robust inference of microbial ecological networks. *PLOS Comput. Biol.* **11**(5): e1004226.

R Core Team (2023). R: A language and environment for statistical computing. R Foundation for Statistical Computing, Vienna, Austria. URL <https://www.R-project.org/>.

Reitmeier, S., Hitch, T., Treichel, N., Fikas, N., Hausmann, B., Ramer-Tait, A. *et al.* (2021). Handling of spurious sequences affects the outcome of high-throughput 16S rRNA gene amplicon profiling. *ISME Commun.* **1,** 31.

Revillini, D., David, A., Menges, E., Main, K., Afkhami, M., Searcy, C. (2022). Microbiome-mediated response to pulse fire disturbance outweighs the effects of fire legacy on plant performance. *New Phytol.* **233,** 2071-2082.

Watts, S., Ritchie, S., Inouye, M., Holt, K. (2019). FastSpar: rapid and scalable correlation estimation for compositional data. *Bioinformatics.* **35,** 1064-1066.
